# Deep Learning-based Inaccuracy Compensation in Reconstruction of High Resolution XCT Data

**DOI:** 10.1038/s41598-020-64733-7

**Published:** 2020-05-06

**Authors:** Emre Topal, Markus Löffler, Ehrenfried Zschech

**Affiliations:** 1 Technische Universität Dresden, Dresden Center for Nanoanalysis, Dresden, Germany; 2 Fraunhofer IKTS, Institute for Ceramic Technologies and Systems, Dresden, Germany

**Keywords:** Imaging techniques, Imaging techniques

## Abstract

While X-ray computed tomography (XCT) is pushed further into the micro- and nanoscale, the limitations of various tool components and object motion become more apparent. For high-resolution XCT, it is necessary but practically difficult to align these tool components with sub-micron precision. The aim is to develop a novel reconstruction methodology that considers unavoidable misalignment and object motion during the data acquisition in order to obtain high-quality three-dimensional images and that is applicable for data recovery from incomplete datasets. A reconstruction software empowered by sophisticated correction modules that autonomously estimates and compensates artefacts using gradient descent and deep learning algorithms has been developed and applied. For motion estimation, a novel computer vision methodology coupled with a deep convolutional neural network approach provides estimates for the object motion by tracking features throughout the adjacent projections. The model is trained using the forward projections of simulated phantoms that consist of several simple geometrical features such as sphere, triangle and rectangular. The feature maps extracted by a neural network are used to detect and to classify features done by a support vector machine. For missing data recovery, a novel deep convolutional neural network is used to infer high-quality reconstruction data from incomplete sets of projections. The forward and back projections of simulated geometric shapes from a range of angular ranges are used to train the model. The model is able to learn the angular dependency based on a limited angle coverage and to propose a new set of projections to suppress artefacts. High-quality three-dimensional images demonstrate that it is possible to effectively suppress artefacts caused by thermomechanical instability of tool components and objects resulting in motion, by center of rotation misalignment and by inaccuracy in the detector position without additional computational efforts. Data recovery from incomplete sets of projections result in directly corrected projections instead of suppressing artefacts in the final reconstructed images. The proposed methodology has been proven and is demonstrated for a ball bearing sample. The reconstruction results are compared to prior corrections and benchmarked with a commercially available reconstruction software. Compared to conventional approaches in XCT imaging and data analysis, the proposed methodology for the generation of high-quality three-dimensional X-ray images is fully autonomous. The methodology presented here has been proven for high-resolution micro-XCT and nano-XCT, however, is applicable for all length scales.

## Introduction

**Figure 1:**
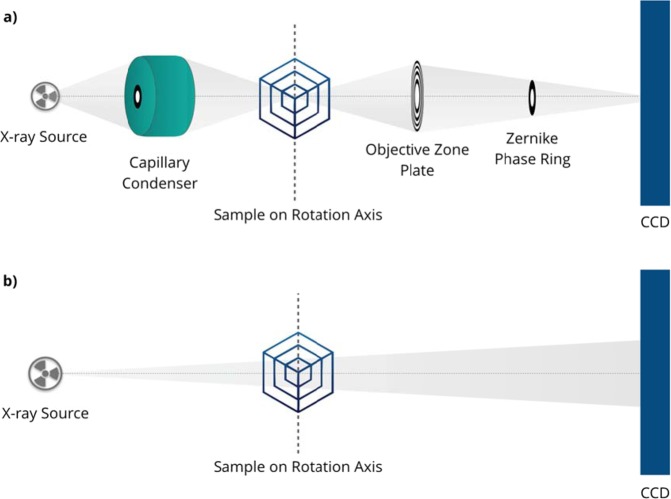
The schematics of (**a**) cone-beam and (**b**) parallel-beam CT geometries.

High-resolution XCT (HR-XCT) using laboratory sources, i.e. micro-XCT and nano-XCT, enables to study objects with micron resolution and even below. Micro-XCT uses a cone-beam projection geometry for imaging of object features of some microns (best resolution 0.7 μm resolution)^1^. Full-field X-ray microscopy and respective nano-XCT use a parallel-beam geometry and Fresnel zone plates as X-ray objective lenses to achieve resolutions down to 50 nm^2^. The schematics of cone-beam and parallel-beam XCT geometries are provided in Fig. 1. During the previous decade, HR-XCT has been increasingly applied to materials science and engineering, particularly for imaging of the morphology of composites and skeleton materials but also for 3D microstructure analysis^3–5^. Compared to other 3D imaging techniques such as focused ion beam (FIB) serial cutting and subsequent scanning electron microscopy (SEM) imaging of the cross-sections as well as electron tomography in the transmission electron microscope (TEM), HR-XCT is non-destructive. Consequently, *in-situ* and operando studies of materials can be performed during thermomechanical loading^6^ and during chemical processes^7^ as well as in several environment^8^.

During the data acquisition time, the objects can move, and consequently, the position of features (relative to the X-ray beam) can vary between adjacent projections. Particularly for image acquisition with high resolution, mechanical instabilities of the sample holder and - in case of X-ray microscopy - of the X-ray optics, sample drift as well as inaccuracy in detector positions introduced by defective and/or underperforming detector elements cause errors in the reconstruction process and result in severe artefacts^9^. Conventional XCT reconstruction algorithms assume a static object and an ideal X-ray beam path, i.e., they do not consider thermomechanical instabilities of tool components and object resulting in motion^10^. This assumption is not fulfilled in many clinical applications due to motions of the patient, and it is violated in technical applications due to motions of sample holder, optics (in case of X-ray microscopy) and/or object, which becomes particularly critical for HR-XCT. Eventually, these issues lead to inconsistencies of the obtained 2D projections and they provide streaking and blurring artefacts in the 3D reconstruction. The quality of data reconstruction depends on amplitude and frequency of the motions of tool components and object relative to the X-ray beam. In order to obtain precise and reproducible information of the internal structure of objects and materials from HR-XCT studies, errors introduced during the image acquisition process have to be eliminated or at least mitigated. Since it is practically impossible or at least costly to avoid these inaccuracies by tool improvements and precise adjustment, a computational methodology is needed to “improve” the raw data. Usually, the initial step is to detect and/or to estimate the motions of tool components and object, and subsequently, to compensate these motions by a reconstruction approach that takes into consideration these estimated/detected motions. However, the majority of methods require additional signals and/or measures to estimate the motion e.g. of the ECG signal in cardiovascular imaging^11^ or fiducial markers^12,13^. The last-mentioned approach uses detection and tracking of markers that are attached to the sample and that are located within the projection images. However, this is not the most convenient approach for HR-XCT due to the limited size of the sample. Entropy-based correction^14–17^ and frequency-based correction^18^ are further approaches that have been reported to address this issue. The entropy-based correction uses the entropy as measure for the reconstruction quality. In order to mitigate the motion effect, the entropy is minimized by iteratively reconstructing with 2D data acquired at several angles during sample rotation^19^. Similarly, the frequency-based correction uses mathematically formulated data consistency conditions to estimate the motion in the frequency domain of a sinogram^20–22^. In this case, the Fourier consistency condition is used to estimate the motion in the sinogram domain, mitigating the 3D motion by a cost function considering 2D detector translations^23^. Epipolar Consistency Condition, another proposed approach, takes advantage from the epipolar geometry of two pinhole cameras using Grangeat’s theorem and Beer-Lambert law. This approach defines a consistency metric between an arbitrary pair of projection images to estimate the motion^24–27^. Neural networks, developed in the 1950s not long after the emergence of Artificial Intelligence research, attempted to simulate the way the brain works though in a greatly simplified form^28^. With the improvements in numerical algorithms and the availability of increasingly powerful computers more and more layers of virtual neurons can be modeled. This progress enables deep learning, and especially neural networks, to beat other machine-learning techniques in image processing and pattern recognition^29–31^. In the purest form, a neural network is a program that maps out a set of virtual neurons and that assigns random numerical values, or “weights”, to connections between them. These weights determine how each simulated neuron responds - with a mathematical output between 0 and 1 - to a digitized feature. If the network does not accurately recognize a particular pattern, a numerical algorithm will adjust the weights. This is the way artificial neural networks can train themselves to recognize complex patterns. In the era of big data, inevitable, deep learning applications have been extended to the field of X-ray computed tomography to provide solutions to issues like artefact reduction and missing data: One attempt of solving the XCT image reconstruction problem is the mapping of filtered back projections identically onto a deep neural network architecture^32^. The use of deep neural networks allows to add different compensation layers for several types of artefacts such as missing data, beam hardening and scatter compensation by end-to-end training. Analogously, the deep neural network can be adapted as post-correction approach subsequently to the reconstruction step. Zhang and Yu suggested a convolutional neural network by training the network using a large database of reconstructed images resulting in a significantly improved reduction of beam hardening artefacts^33^. Another study applied a similar approach to limited-angle tomography. The missing data artefacts were reduced by training the convolutional neural network to extract and to suppress implicit features of artefacts from reconstructed images^34,35^. Neural networks were also used successfully for noise reduction, which results in shorter image acquisition times while retaining the expected internal structure information of an object^36–40^.

This paper presents a fully automated methodology that effectively suppresses artefacts caused by systematic and random errors. Such artefacts can result e.g. from motions of tool components and object during image acquisition in HR-XCT. In order to obtain high-quality reconstruction data, a reconstruction software package is developed that employs the following well known reconstruction algorithms: FBP (Filtered-back-projection)^41^, FDK (Feldkamp-Davis-Kress)^42^, SIRT (Simultaneous Iterative Reconstruction Technique)^43^, MLEM (Maximum Likelihood Expectation-Maximization)^44^, SQS (Separable Quadratic Surrogates)^45^, ART (Algebraic Reconstruction Technique)^46^, DART (Discrete Algebraic Reconstruction Technique)^47^ and SART (Simultaneous Algebraic Reconstruction Technique)^48^. In addition, artefact suppression tools for tool component and object motions, center of the rotation misalignment, detector offset, beam hardening as well as missing data is developed and applied, for both parallel-beam and cone-beam XCT geometries. In order to compensate motion, a novel computer vision methodology coupled with a deep neural network approach is developed which provides estimates for motion by tracking features through adjacent projections. Data recovery from an incomplete dataset is achieved by employing the deep convolutional neural network approach to obtain a new set of 2D projection images instead of suppressing artifacts in the final reconstructed images. The proposed reconstruction methodology is compared with the commercially available software using Signal-to-Noise Ratio (SNR), Peak Signal-to-Noise Ratio (PSNR), Structural Similarity Index (SSIM), Root Mean Square Error (RMSE) and Mean Absolute Error (MAE). Furthermore, the effect of each component of the correction module is investigated, and a quality assessment of these reconstructed images is performed using a blind/reference-less image spatial quality evaluator (BRISQUE).

## Results

**Figure 2:**
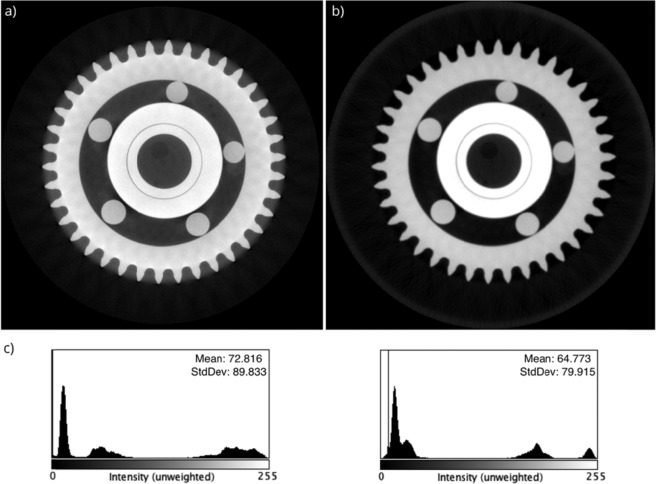
The reconstruction results of the ball bearing sample. Both images are reconstructed with the FDK algorithm using 3200 projections with 2048 × 2048 pixels. The voxel size of the reconstructed images is 3.7 µm. (**a**) commercial reconstruction software, (**b**) customized reconstruction software, and (**c**) respective histograms of reconstructed images.

The main motivation for the development of the reconstruction algorithm and the respective in-house software is to improve the reconstruction quality, and ultimately, to suppress all correctable artefacts. The reconstruction quality of this customized software is compared with a commercially available software of Xradia Inc., which includes user-defined beam-hardening correction, detector offset correction and thermal drift correction, for a special example, a ball bearing. The micro-XCT measurements were performed using a Versa-520 micro-XCT tool of Carl Zeiss (Germany) with a maximum photon energy of 160 keV and a power of 10 W (target current 62 μA). The projections were collected using a polychromatic X-ray source with HE6 source filter at 2301 views with an angular spacing of 0.11° through a detector array of 2048 × 2048 with a pixel size of 0.13 µm. The reconstructed image array is 2048^3^ with a voxel size of 3.7 µm. The reconstruction results are shown in Fig. 2. With our customized software, the total computational time for 3D reconstruction including all the correction steps was about 73 minutes using a computer with Intel Xeon E5-2643 dual CPU, Nvidia Quadro K5000 single GPU and 160 GB RAM.

As shown in the results from exactly the same FDK^42^ algorithm, our customized software outperformed the commercial software. To proof this statement which is based on a visual impression and to validate the numerical approach described in this paper, the reconstruction performance of the commercial software and the customized software was compared based on the PSNR values as shown in Table 1. The PSNR value is an expression for the ratio between the maximum value of a signal and the power of distorting noise that affects the quality of its representation. It is 12.7% higher for the customized software compared to commercial software, which demonstrates the power of including neural network approaches. Furthermore, the customized software achieved to produce lower RMSE and MAE values which means a smaller error compared to the commercial software. Both software packages generated similar results for SSIM.

**Table 1:** The quantitative comparison between reconstruction performance of commercial software and customized software with the ground truth.

Comparison	SNR	PSNR	SSIM	RMSE	MAE
Commercial software/ground truth	6.33	12.51	0.99	54.90	35.93
Customized software/ground truth	7.91	14.10	0.99	45.73	33.15

Signal-to-Noise Ratio (SNR), Peak Signal-to-Noise Ratio (PSNR), Structural Similarity Index (SSIM), Root Mean Square Error (RMSE) and Mean Absolute Error (MAE).

**Figure 3:**
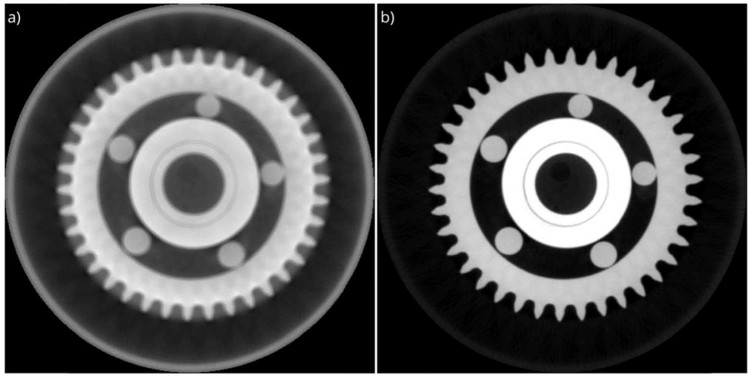
The reconstruction results of the ball bearing using the customized reconstruction software: (**a**) The image is reconstructed using the standard FDK algorithm. (**b**) The image is reconstructed using the FDK algorithm with the proposed correction methodology.

The customized software package is used for the reconstruction of the ball bearing dataset and the reconstructed images with and without applying the proposed methodology is shown in Fig. 3. Ultimately, to understand the contribution of each component to the improvement of the reconstructed image quality, the same dataset is used to explain the effect of each component of the proposed correction methodology separately in the following sub-sections.

### Center of rotation and offset of detector correction

**Figure 4:**
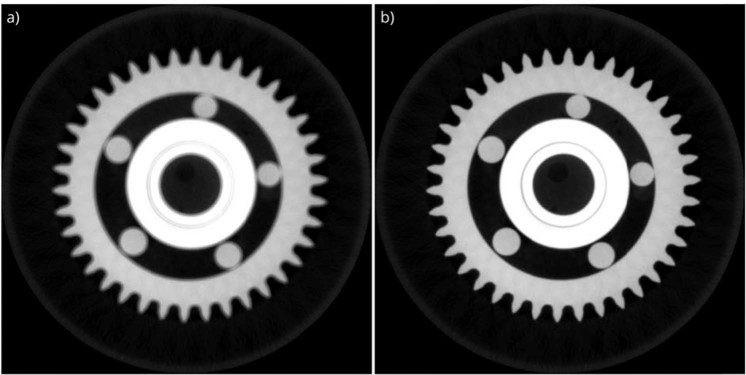
The reconstruction results of the ball bearing. For the reconstruction, the center of rotation and the detector offset correction modules are (**a**) disabled, and (**b**) enabled.

The doubling effect caused by center or rotation and offset of detector misalignments deteriorates the quality and the accuracy of reconstruction. In order to suppress the artifacts and improve the quality and the accuracy of reconstructed image, we applied total variation minimization with the gradient descent algorithm. The result for the center of rotation and the detector offset corrections is shown in Fig. 4. The center of rotation and the detector offset are calculated as 5.1 μm and −190.8 μm, respectively. These corrections suppressed the doubling effect effectively. That means, the applied novel methodology provides a robust way to remove detector misalignment artefacts. The smaller BRSISQUE score indicates a better perceptional quality^49^. The correction improves the quality metric value from 45.7 to 41.6.

### Motion compensation

**Figure 5:**
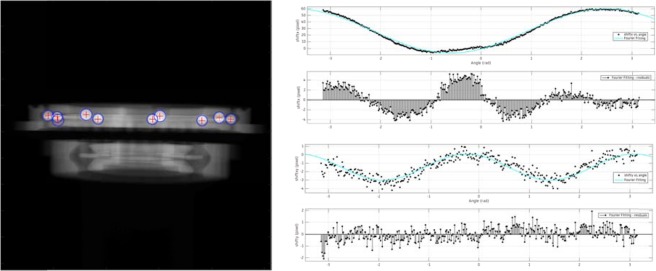
The drilling holes in the ball bearing are used to calculate the motion. The centroid positions of these features are tracked for several rotation angles of the sample. During this procedure, the positions of features are recorded even though they are not available at every projection angle during data acquisition. Once the procedure is completed, one term Fourier fitting is applied to translate the centroid positions to residual shifts from the ideal position of the center beam.

In order to estimate the motion of the object, a computer vision methodology coupled with a deep neural network approach is applied. The procedure of object detection and tracking is illustrated in the projection data of the ball bearing in Fig. 5. Since the data from ball bearing were not used as part of training and validation datasets, they are benchmarks characterizing the performance of the trained network. The centroid positions of tracked features are fitted into the center the X-ray beam path using the one term Fourier model. The R^2^ values of the model in x and y directions are 0.99 and 0.79, respectively, indicating a good fit with the experimental data.

**Figure 6:**
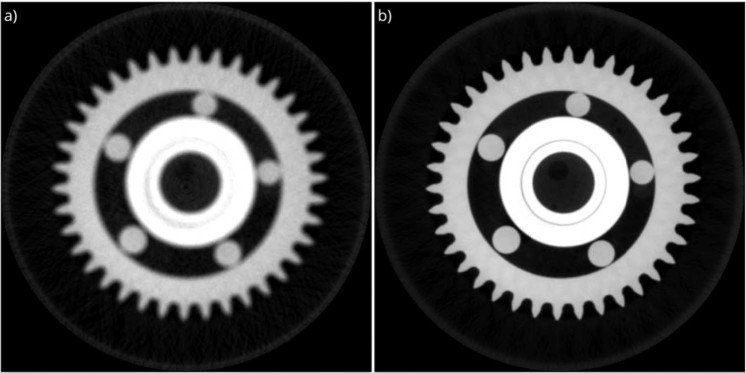
The reconstruction results of the ball bearing. For the reconstruction, the motion compensation module is (**a**) disabled, and (**b**) enabled. The motion is estimated applying the proposed deep-learning based feature tracking method, and the image is reconstructed by considering calculated shifts on the detector plane.

The obtained residual deviations from the ideal X-ray beam path are translated into shifts of the detector plane. These shifts are corrected in the back-projection step of reconstruction. The result of motion compensation is shown in Fig. 6. The developed region-based convolutional neural network is successfully tracking the features available on the projection data. The applied novel motion estimation and compensation methodology removes blurring artefacts and significantly improves the quality of the reconstruction by rearranging detector coordinates according to obtained motion shifts. The proposed method improves the BRISQUE quality metric value from 55.2 to 41.6.

### Missing data recovery

**Figure 7:**
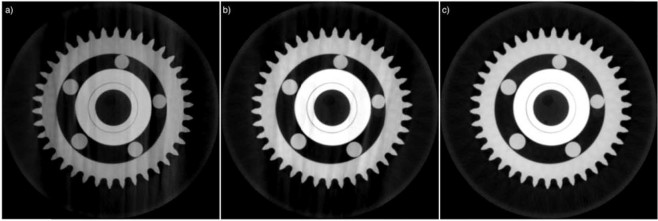
The reconstruction results of the ball bearing. The images are reconstructed with (**a**) projection data with angular coverage of 180°, (**b**) corrected projections with the proposed methodology (same input data as for a), and (**c**) projection data with angular coverage of 360°.

In order to obtain a high-quality image reconstruction, a deep convolutional neural network is used to suppress missing data artefacts on the reconstructed image, and eventually to get high-quality 3D reconstructed data from an incomplete set of projections. This novel methodology is applied to the identical ball bearing data for several data sets, e.g., for an angular coverages of 360° and 180°, respectively. Again, the data acquired from the ball bearing sample were not used for training or validation, and thus, it benchmarks the performance of the trained network. The as-detected and the artifact-corrected projections are compared in Fig. 7. This comparison shows that the proposed methodology effectively suppressed the missing data artefacts that are visible as streaks causing uneven intensity dissipation. The use of corrected projections within the proposed methodology increased the quality metric score from 45.8 to 43.1. Thus, the performance of the applied training model is proven by this validation test.

## Discussion

A fully automated reconstruction methodology and a respective software package were designed and developed to obtain a high-quality reconstruction of high-resolution X-ray computed tomography data, applicable for cone-beam and parallel-beam geometries. The results demonstrate that it is possible to suppress artefacts caused by thermomechanical instability of the tool components and object motion, by center of rotation misalignment inaccuracy in detector positions as well as by incomplete sets of projections. The proposed methodology uses a combination of available software routines and novel solutions in order to improve the quality of reconstruction data with reasonable computational efforts. For the ball bearing sample, the total computation time for 3D reconstruction including all the correction steps was ~73 minutes using a single GPU. The software is developed for multiple GPU, and thus, the computation time can be further reduced using multiple GPUs. Deep learning algorithms were used to enable robust feature tracking in the projection data. Compared to the fiducial marker approach, the novel methodology provides an advantageous alternative to suppress effects of misalignment and object motion. For the example used in this study, the ball bearing, the proposed methodology improved the BRISQUE score remarkably by 13.6 points. The robustness of the developed region-based convolutional neural network was proven for different features. Since multiple features were tracked simultaneously at each given acquired projection image, the accuracy of the detection was validated at the end of each step to eliminate the risk of missing and/or faulty data points. Missing data recovery was demonstrated too, using a novel convolutional neural network methodology. The proposed method effectively suppressed the missing data artefacts by using convolutional neural network (CNN) -generated projections.

## Methods

### Software architecture

**Figure 8:**
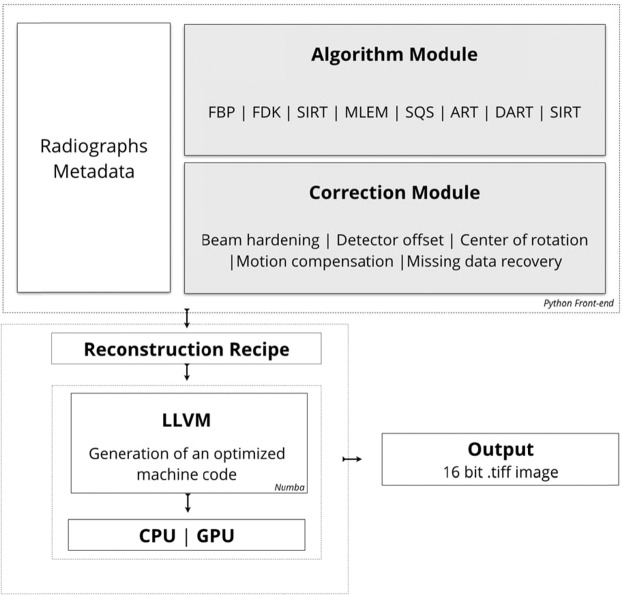
The framework of the software package. The package consists of different modules of algorithms and correction tools which eventually allows users to test several options without compromising the modularity.

The general architecture of the software package is shown in Fig. 8. The Python programming language was selected for the software development since it offers a free, open-source, modular, readable and manageable framework. Furthermore, Python offers an easy integration to low-level programming languages like C and CUDA (Compute Unified Device Architecture). Numba is used as LLVM (Low Level Virtual Machine) compiler infrastructure in order to compile the Python syntax to machine code. Numba supports CUDA GPU programming by directly compiling a restricted subset of Python code into CUDA kernels^50^ and device functions following the CUDA execution model that reduces the computation time significantly.

The software package uses a modular architecture which enables to control the entire process by checking the quality of data analysis at each individual step. The entry point of the reconstruction pipeline is loading the metadata that include information about the data acquisition conditions and the projection data. Once the data and the corresponding acquisition angles are imported, the intensities of raw data are normalized with dark and white field correction, and minus logarithm operation. The reconstruction of these input data is performed applying one of the available algorithms in the algorithm module and subsequently of the available artefact compensation tools in the correction module.

The default approach of the methodology presented in this paper is using a computationally less demanding FDK^42^ algorithm in cone-beam geometry and an FBP^36^ algorithm in parallel beam geometry for data reconstruction, and it is coupling them with the powerful correction module for improving the quality of the resulting reconstructed data. In order to enable a reproducible correction, a voxel-based bilinear interpolation approach is applied for the back-projection step which enables to tailor voxel positions according to pixel positions.

### Center of rotation and offset of detector correction

In order to suppress ring artefacts introduced by the offset of the detector, the inaccuracy of the detector position has to be taken into consideration during the reconstruction step. We applied a reconstruction-based total variation minimization method with a multi-range testing approach to estimate the detector offset and the center of rotation. The total variation of reconstructed images is used as a measure for the quality of the resulting data, and the optimization of this function is fulfilled using the gradient descent algorithm^51–54^. The total variation is the sum over all pixels of squared differences from neighboring pixels. Given an image with *n* × *m* pixels, and *I(x, y)* as the intensity of the pixel *(x, y)*, the total variation is defined as Eq. (1):

1 $$TV(I)=\mathop{\sum }\limits_{y=1}^{m}\mathop{\sum }\limits_{x=1}^{n}|I\text{'}(x,y)|.$$

For a differentiable function *F(u,v)*, the gradient descent recursion with *k* iterations is defined as Eq. (2):

2 $$({u}^{i+1},{v}^{i+1})=({u}^{i},{v}^{i})-{\alpha }_{i}\,{F}^{\text{'}}({u}^{i},{v}^{i}),$$

where 1 < *i* < *k*, ${\alpha }_{i}$ is the step size of the *k*^*th*^ iteration, and *u* is center of rotation parameter, *v* is offset of detector parameter, and ${F}^{\text{'}}({u}^{i},\,{v}^{i})$ is:

3 $${F}^{\text{'}}({u}^{i},{v}^{i})=\,{\left(\frac{\delta {F}^{2}}{\delta {u}_{1}\delta {v}_{1}},\frac{\delta {F}^{2}}{\delta {u}_{2}\delta {v}_{2}},\ldots ,\frac{\delta {F}^{2}}{\delta {u}_{n}\delta {v}_{n}}\right)}^{T}.$$

**Figure 9:**
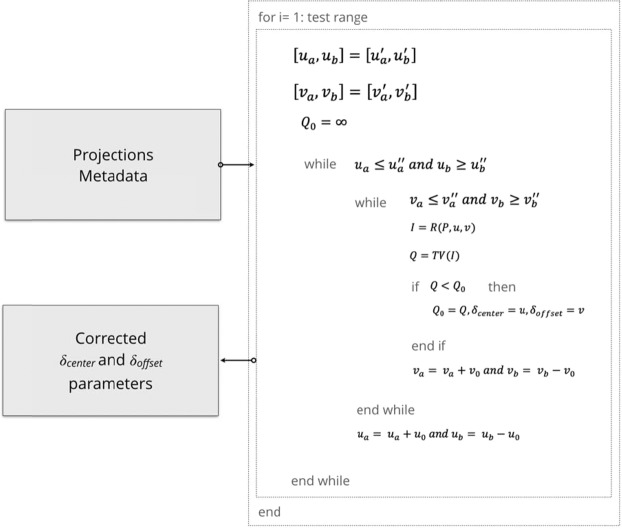
The implementation of the algorithm for the center of rotation and the offset of detector corrections. Subscripts *a* and *b* defines the range, and 0 means the initial point for the recursion.

We can replace ${F}^{\text{'}}({u}^{i},\,{v}^{i})$ with total variation operator $TV{(R(P,u,v))}^{\text{'}}$ in Eq. 2:

4 $$({u}^{i+1},{v}^{i+1})=({u}^{i},{v}^{i})-{\alpha }_{i}TV{(R(P,u,v))}^{\text{'}}({u}^{i},{v}^{i}),$$

where *R* is the reconstruction operator and *P* is the projection. As *i* approaches to *k*, gradient descent recursion approaches toward the minimum and the least total variation yields the corrected parameters. The algorithm describing the applied methodology is given in Fig. 9.

Considering the size of typical HR-XCT data sets, the 2D projection images are initially downscaled by binning to increase the overall performance of the algorithm. After the initial optimization, the corrected parameters are upscaled and further optimized with the same approach with a fine-tuned range, and eventually used as final reconstruction parameters for center of rotation and offset of detector.

### Motion compensation

**Figure 10:**
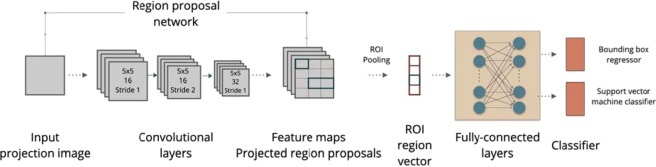
The architecture of a R-CNN + SVM used for feature detection and tracking. All the convolutional layers use 5 × 5 kernels and ReLU as activation functions.

The approach used for motion estimation is based on the fiducial marker approach^12,13^, however, detection and tracking purely relies on intrinsic patterns of the object on two adjacent acquired 2D projections. Computer vision methodologies have been developed for various applications, from cancer diagnosis to defect inspection, which require high efficiency and sensitivity to detect features in the interior of the objects in 2D and 3D^55^. However, the main drawback of the direct application of computer vision methodologies in computed tomography reconstruction, particularly for HR-XCT, is the change of the features detected and tracked in the acquired projections over time, e.g. every single projection provides an individual view of the object. In order to overcome this obstacle, a robust feature detection and tracking methodology that considers rotation and change of shapes of detected features is required. Consequently, we combined computer vision and deep neural network approaches to detect and to track features throughout the adjacent projections. A region-based CNN (R-CNN) is used for extracting feature maps for detection of intrinsic patterns/features from acquired projections and giving a unique id to each of them. The network design is completed with addition of a Support Vector Machine (SVM) classifier to provide a robust tracking over the entire projections. The proposed R-CNN+SVM architecture for feature detection and tracking is illustrated in Fig. 10.

The CNN used for the model is a standard deep-CNN with 3 convolutional layers and two fully-connected layers. Each convolutional layer uses a 5 × 5 kernel, and a rectified linear unit (ReLU) is used as activation function. The CNN part extracts feature maps from input and passes these maps through a region proposal network (RPN)^56^. The RPN in the R-CNN^57^ is used to determine searching positions in order to reduce computational efforts for the overall inference process. The RPN uses a selective search to quickly and efficiently scan possible locations and to discard unlikely feature positions by outputting bounding box proposals with scores representing the probability of containing the object or not at each location. An SVM classifier is used to classify and to decide whether the predicted bounding box include the feature or not. The output of the model includes the centroid coordinates of detected features and their respective id numbers.

**Figure 11:**
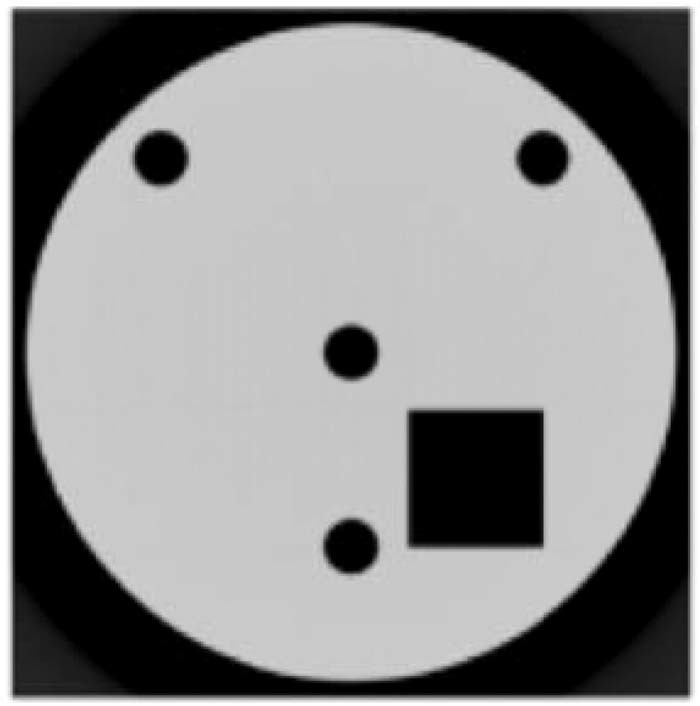
An example of simulated phantoms used for training of R-CNN.

The model training is performed by adapting an unsupervised training approach. The training data used for the model is generated through forward projections of simulated phantoms including several simple geometrical patterns such as sphere, triangle and rectangular which might be easily encountered in real tomographic datasets. The sample size of generated dataset was consisted of 3600 projection images. An example of used phantoms is given in Fig. 11. Finally, the feature maps extracted applying the CNN part are used to train the SVM part. R-CNN is optimized or a multi-task loss function which combines the losses of classification and bounding box regression. During the training, we found that smaller learning rates converge faster and thus we used a learning rate of 0.001.

**Figure 12:**
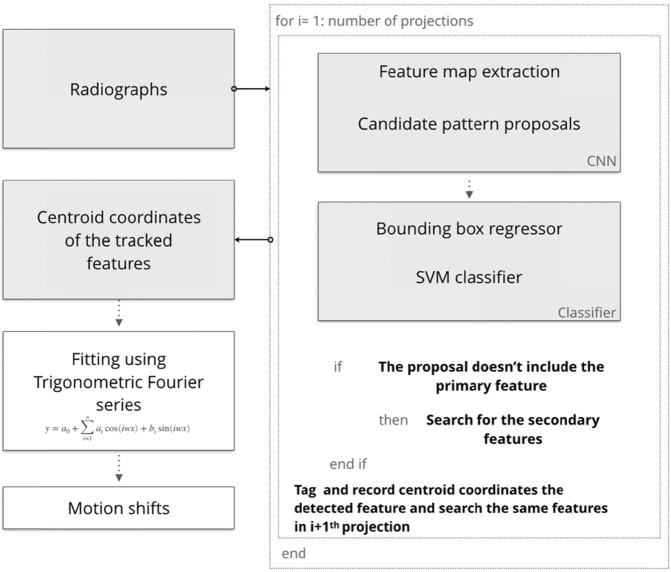
The algorithmic workflow of proposed feature detection and tracking method.

The algorithmic implementation of the proposed methodology is given in Fig. 12. Each individual projection is loaded to the trained model as input, and the outputs are recorded. Once the detection and tracking task is completed for each projection, the motion compensation module combines the outputs - feature id numbers and respective centroid coordinates - and calculates the confidence score of each id which simply provides a rate of how many times the same feature is detected until the entire angular range is covered. The respective coordinates of feature id with highest confidence score then used for further steps. The coordinates of the central point of angular coverage are used as a reference point, and subsequently, the absolute difference of the coordinates from this reference point is calculated for each angle. Then, these absolute difference values are fitted into Fourier series, i.e. a sum of sine and cosine functions that describe a periodic signal, to obtain the position of the center of the X-ray beam path. The outcome of this fitting step are the motion shifts in the detector plane for each projection angle. Ultimately, the motion compensation module transfers the obtained motion shifts to geometry metadata which provide rearranged detector positions for the back-projection step in order to compensate the motion effect.

The proposed methodology eliminates the dependency of object and feature tracking on fiducial markers, and it provides a powerful and adaptable way to determine the motion of the object. Furthermore, it is applicable for both parallel-beam and cone-beam geometries.

### Missing data recovery

**Figure 13:**
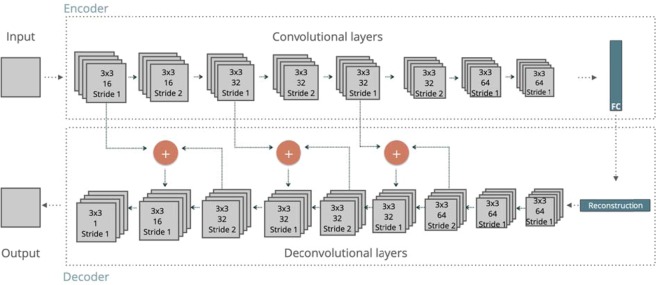
The architecture of the convolutional neural network for data recovery. All the convolutional and deconvolutional layers use 3 × 3 kernels and ReLU as activation functions.

To infer high-quality reconstruction data from incomplete sets of projections, we used a deep-learning approach which enables a way to understand the transformation between complete and incomplete projections and the angular dependency of the limited angle reconstruction artifacts. A deep convolutional neural network (CNN) was used for extracting feature maps from incomplete set of projections through convolutional layers in the encoder part and deconvolutional layers in the decoder part. The proposed CNN architecture for data recovery is illustrated in Fig. 13. The use of encoder/decoder architectures^58,59^ allows to generate an image as output since the decoder part maps the feature representation generated by encoder part back to input data space.

The encoder part of CNN used in the model is composed of 8 convolutional layers which use 3 × 3 convolutional kernels with numbers changing between 16 and 64, and a fully connected layer. Three of eight convolutional layers use 2 × 2 convolutional stride that computes the convolution from every 2 pixels, in order to reduce the size by half at each. The fully connected layer transfers encoded data with new image size to decoder part. The decoder part of CNN is composed of 9 deconvolutional layers which use 3 × 3 convolutional kernels with numbers changing between 1 and 64. This time, 2 × 2 deconvolutional strides in three deconvolutional layers up-sample the data back to the original input size. The final convolutional layer generates an output image. The feature maps generated from the deconvolutional layers of the decoder part are merged with the preceding feature maps at the same scale generated from convolutional layers of the encoder part. These merged layers are used to avoid the resolution loss during the down-sampling process in the encoder part.

The model training is done by adapting an unsupervised training approach. The complete and incomplete projection set pairs of simulated phantoms (see Fig. 11) with angular coverage of 30°, 60°, 120° and 180° are used as training dataset. The CNN is optimized by using Adam^60^. The learning rate, the exponential decay rate for the first moment estimates, the exponential decay rate for the second moment estimates and epsilon were respectively 0.001, 0.9, 0.99 and 10^−8^. Once the training is performed, the CNN transformation model fits weights of the network to predict a new set of projections by learning the transformation between complete and incomplete projections.

## Data Availability

The data that support the findings of this study are available from the corresponding author upon reasonable request.
